# Supplementary material for: In vivo transplantation of 3D encapsulated ovarian constructs in rats corrects abnormalities of ovarian failure
Source: Nat Commun. 2017 Dec 5;8:1858. doi: 10.1038/s41467-017-01851-3 (PMC5717171; doi:10.1038/s41467-017-01851-3)
Supplement: Supplementary file 1 — Supplementary Information [file 41467_2017_1851_MOESM1_ESM.pdf]

Supplementary figure 1

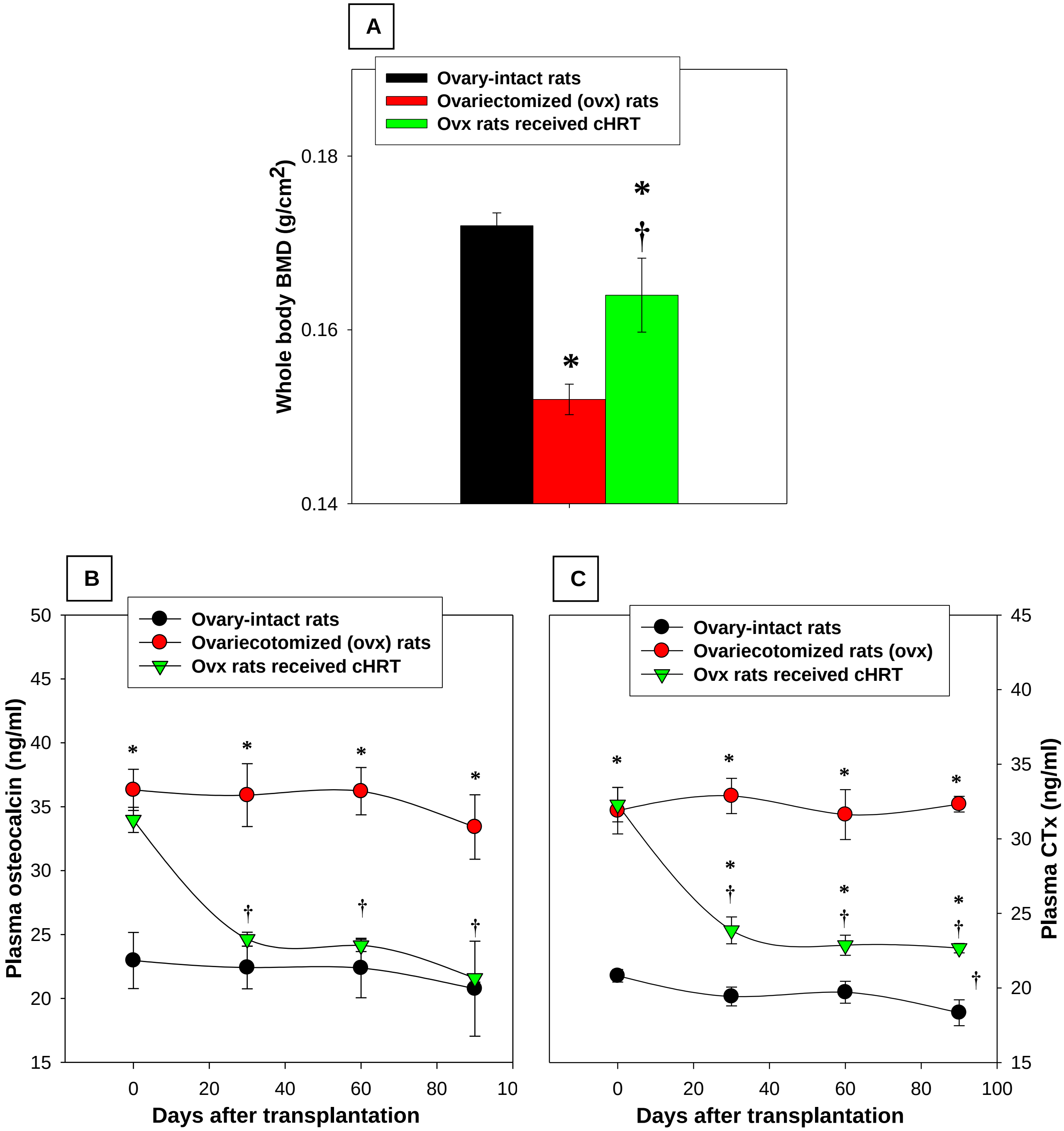

Whole body BMD and Biomarkers of bone metabolism. (A) Quantitative DXA scan (B) plasma osteocalcin and (C) C-telopeptide of collagen levels during the study period of 90 days in ovariectomized rats implanted with ovarian cell constructs compared to ovary-intact rats and untreated ovariectomized groups. Each data point represents mean ± SEM of 10 values. Statistics: one-way ANOVA followed by Student-Newman-Keuls *post hoc* analysis. * indicates significance P<0.05 compared to ovary-intact rats; † indicates significance P<0.05 compared to ovariectomized rats. The figures represent data from one of three separate experiments.

Supplementary figure 2

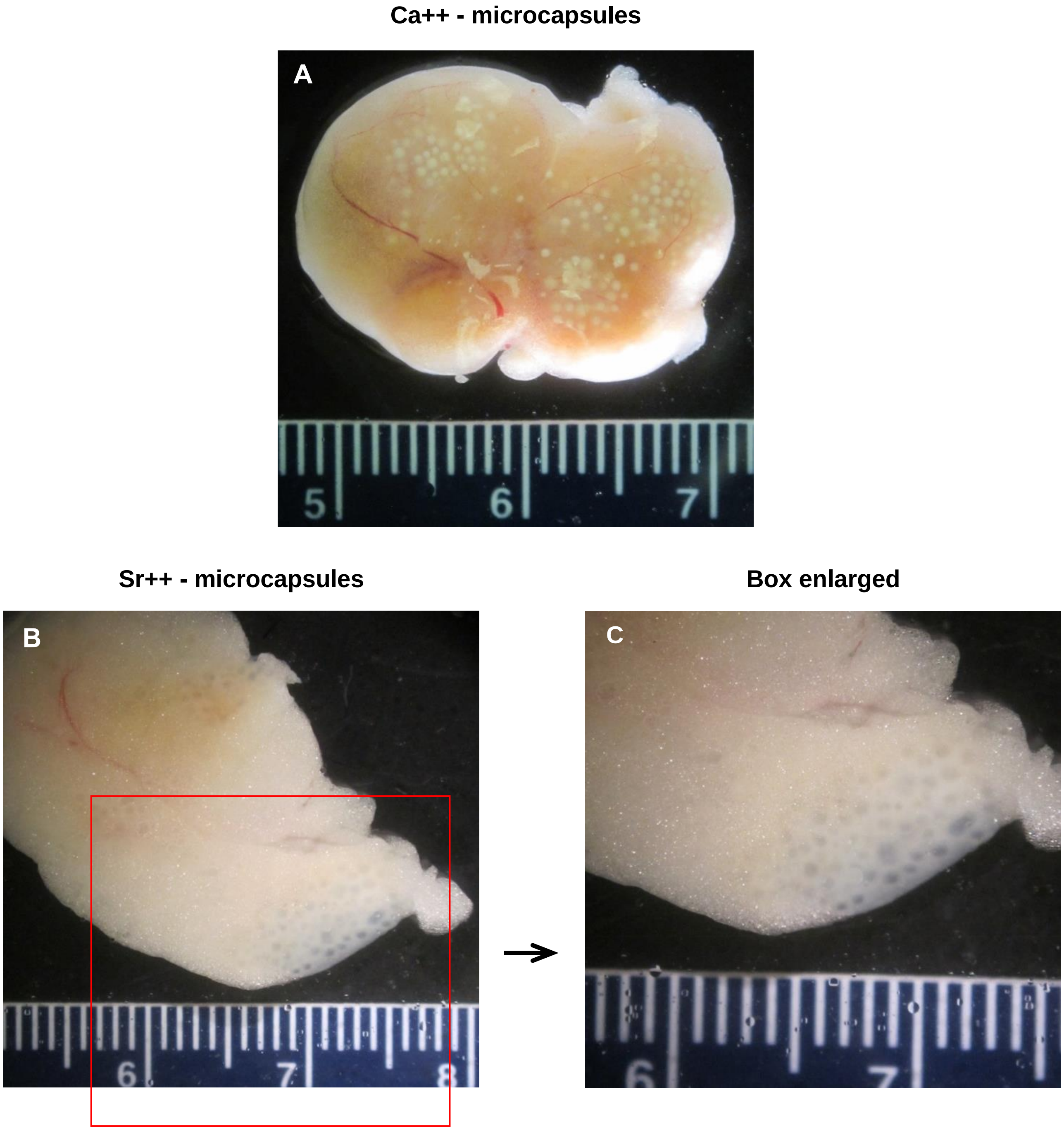

Images of constructs explanted after 90 days in an omental pouch. Explants for constructs using Ca^{++} (**A**) or Sr^{++} (**B**) as crosslinker. Image (**A**) is consistent with the image shown in Figure 1D, where evidence of calcification was observed. However, (**B**) indicates absence of calcification with the use of the Sr^{++} crosslinker. An expanded view of the explants from Sr^{++} crosslinked alginate further evidences the lack of calcification as indicated by the more translucent appearance of the explant (**C**).

Supplementary table 1

	Ovary-intact	Ovx	Ovx+10µg E_2	Ovx+50µg E_2	Ovx+2mg P_4	Ovx+10µg E_2 +2mg P_4	Ovx+50µg E_2 +2mg P_4	Ovx + cHRT
Bone volume fraction (%)	44.85 ± 0.53[a]	14.94 ± 1.69[b]	23.65 ± 0.29[b]	30.55 ± 1.11[c]	16.65 ± 1.35[b]	24.52 ± 1.33[b]	30.89 ± 1.78[c]	32.71 ± 1.01[c]
Trabecular number (1/mm)	4.127 ± 0.04[a]	1.992 ± 0.12[b]	2.903 ± 0.07[c]	3.334 ± 0.09[c]	2.240 ± 0.16[b]	2.953 ± 0.07[c]	3.264 ± 0.09[c]	3.249 ± 0.13[c]
Trabecular thickness (µm)	101.6 ± 0.46[a]	74.33 ± 4.07[b]	81.52 ± 1.52[b]	91.58 ± 1.06[c]	74.28 ± 1.91[b]	82.93 ± 3.14[b]	94.43 ± 3.13[a,c]	91.35 ± 2.86[a,c]
Trabecular separation (µm)	125.1 ± 2.19[a]	432.8 ± 33.2[b]	263.5 ± 7.12[c]	209.1 ± 8.99[d]	379.2 ± 32.5[b]	256.4 ± 10.4[c]	212.6 ± 11.6[d]	206.5 ± 11.6[d]

Restoration of femur bone trabecular micro-architecture in ovx rats by constructs. Each data point represents mean ± SEM of 10 values. Different letters above the values represents statistical significance at $P<0.05$ between the groups. Same letters denote no significant difference between the groups as determined by one-way ANOVA followed by Student-Newman-Keuls *post hoc* analysis. Ovx – Ovariectomized; E_2 – 17β-estradiol; P_4 - progesterone
